# Case Report: Unilateral relapsing primary central nervous system vasculitis—expanding the phenotype

**DOI:** 10.3389/fimmu.2025.1502022

**Published:** 2025-06-02

**Authors:** Turlough Montague, Jenny Han, Emily Cheung, James Drummond, Hwei Choo Soh, Jeannette Lechner-Scott, John Parratt

**Affiliations:** ^1^ Department of Neurology, Royal North Shore Hospital, Sydney, NSW, Australia; ^2^ Department of Neurology, John Hunter Hospital, Newcastle, NSW, Australia; ^3^ Brain Imaging Laboratory, Department of Radiation Oncology, Royal North Shore Hospital, Sydney, NSW, Australia; ^4^ Department of Anatomical Pathology, Royal North Shore Hospital, Sydney, NSW, Australia

**Keywords:** vasculitis, PCNSV, anti CD20 monoclonal antibody, relapsing, unilateral, MRI

## Abstract

**Background:**

Unilateral relapsing primary central nervous system vasculitis (UR-PCNSV) is a scarcely reported subtype of PCNSV. It is characterised by frequent relapses with lesions confined to a single hemisphere. Herein, we expand the phenotype of UR-PCNSV, adding three cases to the existing 13 in the literature.

**Method:**

A retrospective review of clinic databases at two adult tertiary referral centres in New South Wales, Australia, was undertaken to identify cases of UR-PCNSV. Predefined inclusion criteria were (1) biopsy-proven PCNSV, (2) lesions confined to a single hemisphere, and (3) two or more relapses as evidenced by new enhancing lesions on MRI.

**Results:**

Three cases of biopsy-proven UR-PCNSV were identified. All demonstrated three or more relapses with new lesions confined to the same hemisphere. The mean age was 34.5 (± 8.6) years, and the median delay to diagnosis was 12 months (IQR 7.5–21). Headache was the first symptom in all patients, and they developed unilateral motor and sensory deficits. Cognitive impairment was a prominent feature in one and none developed seizures. CT and/or MR angiography showed normal results. MRI head showed both subcortical and cortical lesions with parenchymal and leptomeningeal enhancement. The protein level was normal in all patients, and one had a mildly raised white cell count (9 × 10^9^/L). Biopsy in all three demonstrated a T-cell predominant perivascular lymphocytic infiltrate with areas of transmural inflammation and infarct-like necrosis. Despite treatment with anti-CD20 monoclonal antibodies, relapses occurred after steroid withdrawal in all. Prolonged steroid with additional immunosuppression was required to maintain remission. All patients demonstrated hemiatrophy within 12 months of presentation.

**Conclusion:**

Compared with typical PCNSV, this rare unilateral, relapsing subtype has a younger age of onset, lower prevalence of angiographic abnormalities, and frequent relapses. Our patients had persisting lesion enhancement despite anti-CD20 mAb monotherapy and demonstrated hemiatrophy within the first year, indicating high inflammatory activity and a requirement for additional immunosuppression. This case series additionally highlights the overlapping clinical and radiological features of PCNSV and CNS demyelination, which may contribute to diagnostic delay.

## Introduction

UR-PCNSV is a very rare subtype of PCNSV, with only 13 cases previously reported (1–5). Diagnosis can be challenging as angiographic abnormalities are uncommon, and more data are needed to inform optimal treatment strategies. The addition of this case series supports existing assertions about the entity while expanding the phenotype further.

## Methods

A retrospective review of clinic databases at two tertiary referral centres in New South Wales, Australia, was undertaken. Inclusion criteria were as follows: (1) biopsy-proven PCNSV, (2) lesions confined to a single hemisphere, and (3) two or more relapses as evidenced by new enhancing magnetic resonance imaging (MRI) lesions.

## Case series

Three female patients were identified with a mean age of 34.5 (± 8.6) years. One patient was Vietnamese, and two were Caucasian. One patient had a longstanding history of well-controlled focal epilepsy treated with lamotrigine. The remainder had no relevant past medical history.

**Table 1** summarises the cases. All patients experienced subacute onset headache that worsened over 1 to 4 weeks and unilateral motor and sensory deficits. Patient 1 evolved to dense right-sided hemiparesis and hemisensory loss associated with moderate cognitive impairment. Patient 2 had a hemispastic gait and right-hand apraxia. There were no seizures, and the only reported systemic symptom was fatigue.

**Table 1:** Summary of cases.

		Patient 1	Patient 2	Patient 3
*PAtient bio*	*Age at symptom onset (years)*	46	29	27
	*Gender*	F	F	F
	*Ethnicity*	Caucasian	Vietnamese	Caucasian
	*Follow-up duration (years)*	4	3	1.5
*Clinical features*	*Symptom onset*	Subacute (1 month)	Subacute (2 weeks)	Subacute (1 month)
	*Headache*	Yes	Yes	Yes
	*Seizure*	No	No	No
	*Cognitive impairment*	Yes	No	Yes
	*Motor deficit*	Yes	Yes	Yes
	*Sensory deficit*	Yes	Yes	Yes
	*Treatments & response*	Pulsed CS: clinical and radiological improvement MMF + CS: remission RTX: relapse RTX + CS + MMF: remission	Pulsed CS + PLEX: clinical and radiological improvement Ocrelizumab: relapse CS + MMF: remission	Pulsed CS: clinical and radiological improvement Ocrelizumab: relapse CS + CYC (IV): remission
	*Number of relapses*	4	5	3
	*Clinical outcome (mRS)*	mRS 0 Embouchure’s dystonia	mRS 1 Right leg spasticity	mRS 2 Left hemisensory loss, dysarthria, fatigue
*CSF*		Acellular Normal protein OCB: matched serum and CSF	1 mononuclear 7 RBCs Normal protein OCB -ve	WCC 9 (95% mononuclear, 5% polymorph) 0 RBCs Normal protein OCB -ve
*Radiology*	*Lesion number*	5-10	>10	5-10
	*Lesion distribution*	Left hemisphere Cortical & subcortical	Left hemisphere Subcortical, extensive corticospinal tract brain lesion	Right hemisphere Cortical and subcortical
	*Pattern of enhancement*	Nodular, patchy	Irregular ring, nodular and leptomeningeal	Linear, patchy
	*Diffusion restriction*	Yes	Yes	Yes
	*Microhaemorrhage on SWI*	No	Yes	Yes
	*Vessel abnormality on MRa or CTa*	No	No	No
	*Hemiatrophy*	Yes	Yes	Yes
*ANATOMICAL* *pathology*		Lymphocytic vasculitis: T-cell predominant	Lymphocytic vasculitis; T-cell predominant	Lymphocytic vasculitis: T-cell predominant
*Time from symptom onset to biopsy*		18 months	12 months	10 months

**Figure 1:**
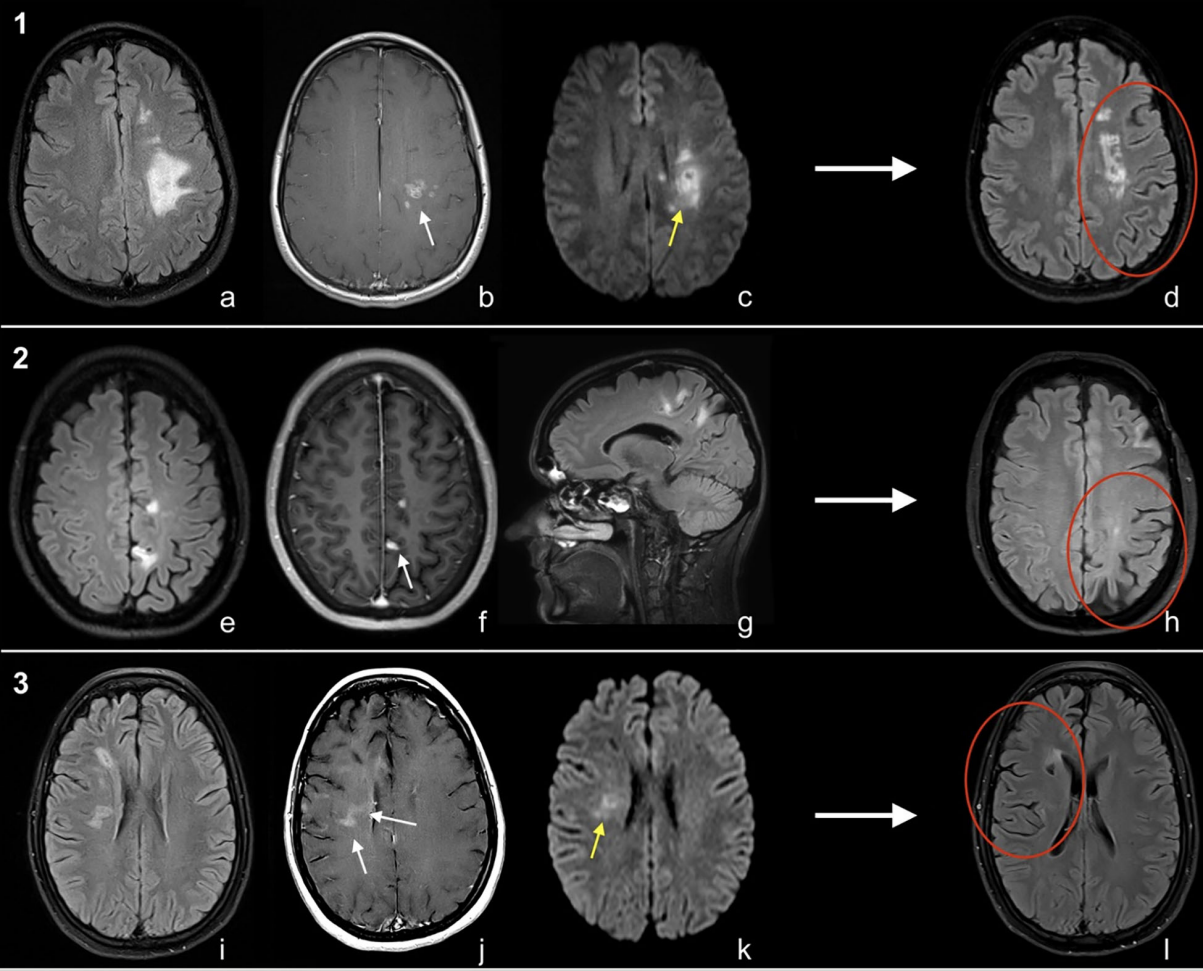
MRI brain of cases [1-3, **(a-l)**]. Patient 1; Ax T2FLAIR **(a)**, Ax T1C+ **(b)** and Ax DWI **(c)**. Patient 2; Ax T2FLAIR **(e)**, Ax T1C+ **(f)**, Sag T2FLAIR **(g)**. Patient 3; Ax T2FLAIR **(i)**, Ax T1C+ **(j)**, Ax DWI **(k)**. Long-term follow-up Ax T2FLAIR (d/h/l) for all patients. Imaging shows regions of enhancement (white arrows), diffusion restriction (yellow arrows), and unilateral atrophy on long-term follow-up MRI (red circles).

MRI brain T2/FLAIR sequences demonstrated multiple unilateral hemispheric lesions, with predominant involvement of supratentorial white matter (**Figure 1**). Patient 2 had over 10 lesions at presentation including a tumefactive left frontal lesion with perilesional oedema. The others had 5–10 lesions in a mostly pericallosal, periventricular, and subcortical distribution. The left hemisphere was affected in two patients and the right hemisphere in one. All showed some diffusion restriction of lesions. Post-contrast T1 sequences showed parenchymal enhancement in all patients and leptomeningeal enhancement in one. Parenchymal patterns of enhancement varied and included irregular incomplete rings and nodular and linear enhancement. On susceptibility-weighted imaging (SWI), there were few, subtle foci of susceptibility artefact (<10 mm) consistent with microhaemorrhage in patients 2 and 3.

CSF results are shown in **Table 1**. Additional serum and CSF testing for immune, infective, and malignant aetiologies was performed, which showed negative results (**Supplementary Table S1**). Patients underwent computed tomography (CT) and/or MR angiography, which did not identify any features of vasculitis. CT neck to pelvis and whole-body fludeoxyglucose-18 positron emission tomography (FDG-PET) scans were done to investigate for malignancy or systemic vasculitis, which was not found.

**Figure 2:**
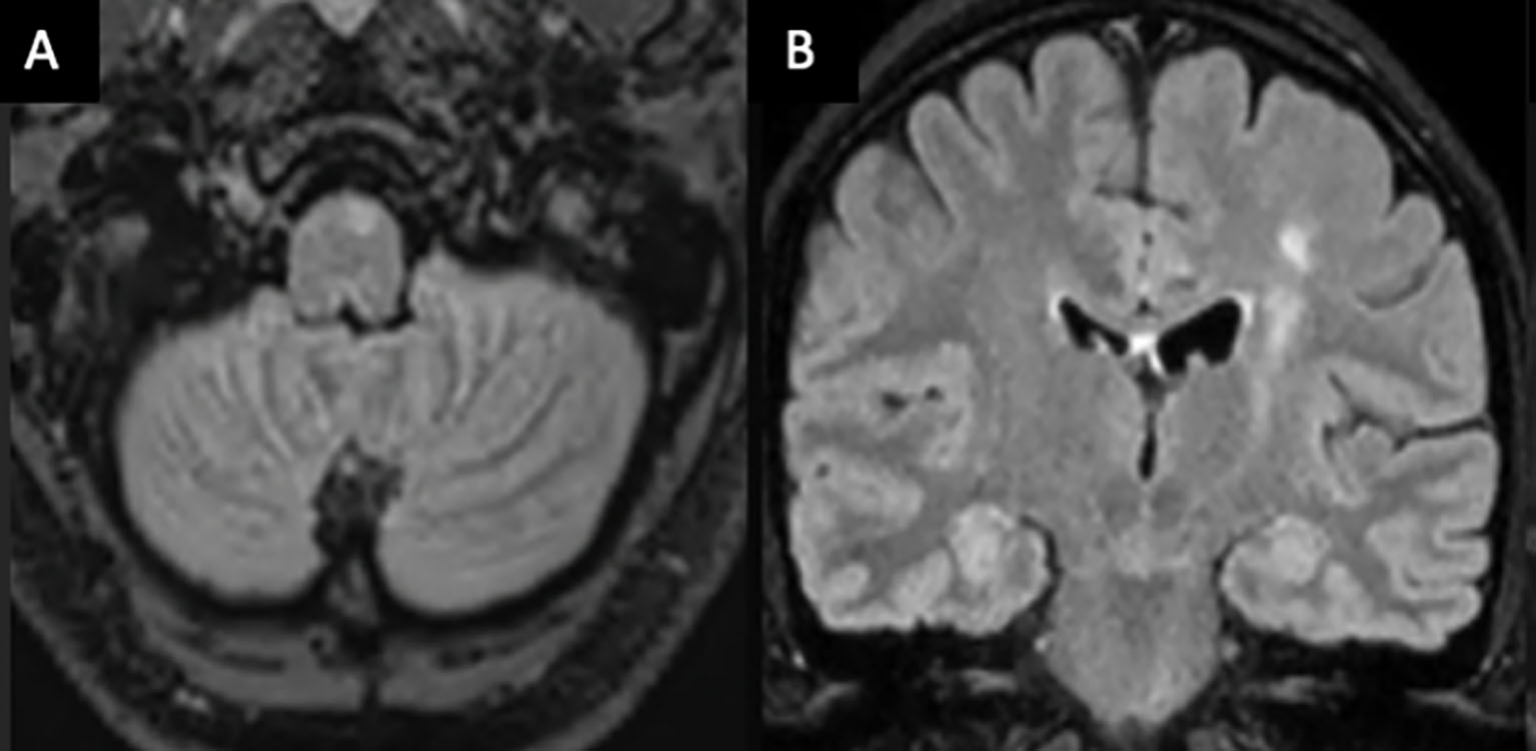
Axial **(A)** and coronal **(B)** MRI brain T2/FLAIR-weighted imaging of patient 2 demonstrating a high T2 signal along the left corticospinal tract from the pre- and post-central gyrus into the ipsilateral medulla, consistent with Wallerian degeneration.

Vessel wall MRI and digital subtraction angiography (DSA) were not performed in patient 1 due to their low sensitivity for small vessel vasculitis (6), and because biopsy would still be required for definitive diagnosis. Patients 2 and 3 were suspected to have CNS demyelinating disease given overlapping clinical and imaging characteristics and commenced ocrelizumab. In patient 2, high signal along the left hemisphere corticospinal tract from the motor cortex to ipsilateral medulla was thought to reflect a demyelinating lesion (**Figure 2**), as reported in neuromyelitis optica spectrum disorder (7). However, serial imaging was most consistent with Wallerian degeneration secondary to a tumefactive lesion involving the left motor strip. Patient 1 was treated for suspected PCNSV with mycophenolate. More potent immunosuppression, such as cyclophosphamide, was considered inappropriate as first line for this patient due to a milder, more insidious disease presentation. All patients received 1 g of intravenous methylprednisolone for 3 days at initial presentation and in patient 1; this was followed by a 3-month tapering course of oral prednisolone.

Despite treatment, each patient had three or more relapses, which were typically associated with recurrence of headache and fatigue. Development of significant new neurological signs was uncommon, although existing deficits transiently worsened. With each relapse, MRI demonstrated enhancement of existing lesions and/or new enhancing lesions. Relapses were treated with additional pulsed corticosteroid. Patients 2 and 3 continued ocrelizumab, whereas patient 1 switched to rituximab. However, after a further 6 months of treatment, relapses continued to occur in all, prompting brain biopsy for diagnostic clarification.

**Figure 3:**
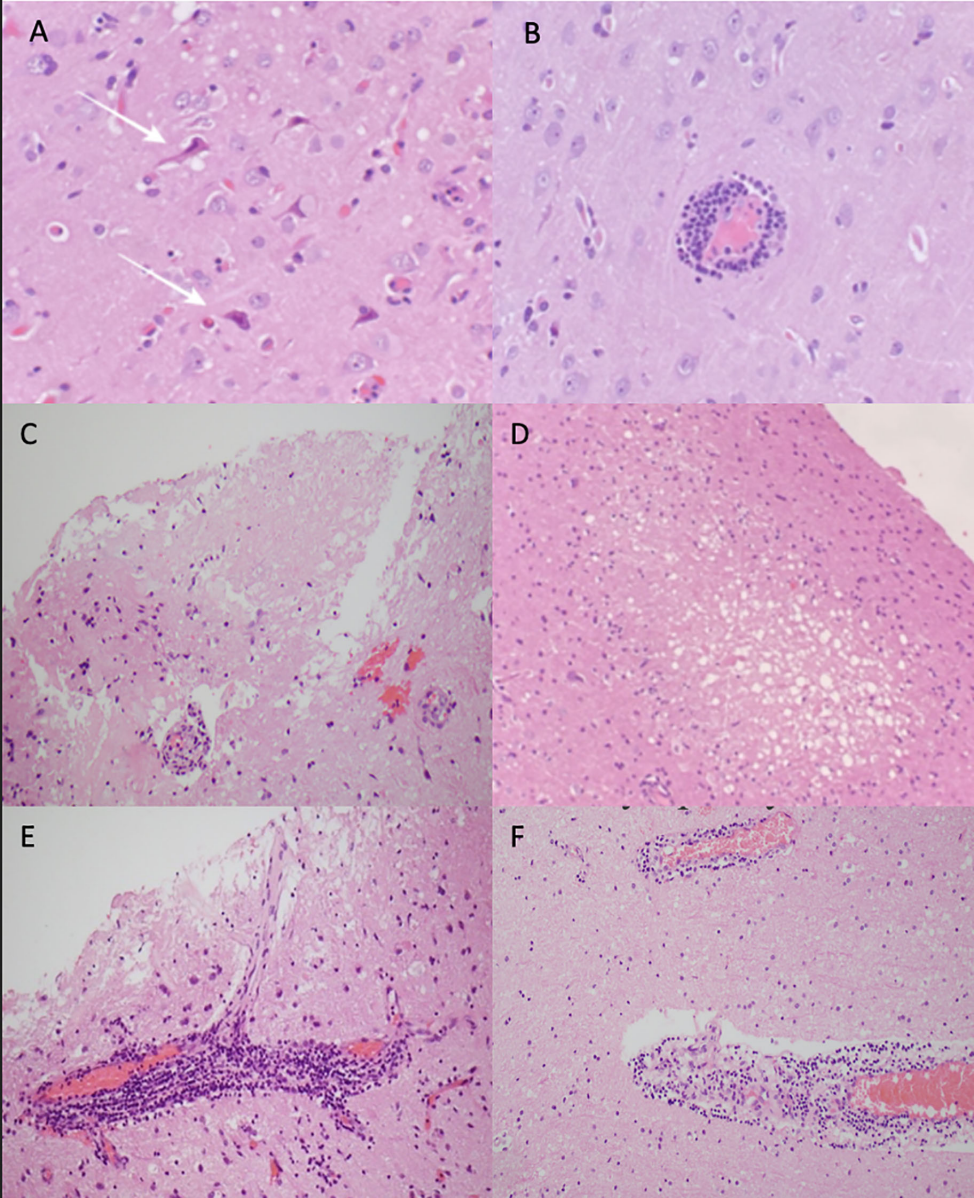
Photomicrographs of haematoxylin and eosin-stained sections showing case 1: **(A)** ischaemic appearing red neurons (white arrows), **(B)** perivascular lymphocytic cuffing around a blood vessel, and **(C)** area of infarct like necrosis. Case 2: **(D)** small foci of spongiosis and necrosis focally in the cortex and **(E)** focally florid perivascular lymphocytic inflammation, which in places infiltrates the vessel wall. Case 3: **(F)** perivascular lymphocytic inflammation.

Photomicrographs of anatomical pathology are shown in **Figure 3**. Histopathology showed small-calibre vessels with perivascular lymphohistiocytic inflammation and transmural vessel inflammatory infiltrate along with endothelial swelling. There were multiple foci of infarct-like necrosis involving both grey and white matter. There were no granulomas, necrotizing features, or glial or lymphoid malignancy. There was no demonstrable demyelination and viral markers (HSV1/2, CMV, SV40), and amyloid and aquaporin-4 immunohistochemistry staining showed normal results. Findings in all were consistent with lymphocytic vasculitis.

T1 hypointense “black holes” developed within lesions over time and within 12 months of symptom onset; all patients demonstrated cerebral volume loss in their affected hemisphere (**Figures 1d, h, i**). Following biopsy confirmation of UR-PCNSV, further treatment changes were made (**Table 1**: treatment and response). Patients 1 and 2 have had no new lesions for 18 and 12 months, respectively, on mycophenolate and 10 mg of prednisolone daily. Patient 1 additionally receives 6-monthly rituximab ongoing. Ocrelizumab has been stopped in patient 2, although CD19+ B cells remain depleted (<0.01 × 10^9^/L) at 10 months since last infusion. Patient 3 commenced monthly intravenous cyclophosphamide along with a tapering course of prednisolone and after 6 months has had no further relapses. The patients have a respective modified Rankin Scale (mRS) of 0, 1, and 2. For patient 1, the only persisting neurological problem is Embouchure’s dystonia, which impacts clarinet playing. Patient 2 has residual right-leg spasticity impacting gait, and patient 3 has persisting left hemisensory loss, dysarthria, and fatigue.

## Discussion

UR-PCNSV patients are younger with a median age at diagnosis of 31.2 (± 15.6) years compared with 48 years in a large PCNSV cohort study (8). No angiographic abnormalities have been found in UR-PCNSV patients, including seven that underwent conventional angiography (1), indicating involvement of only small-medium sized vessels. Comparatively, typical PCNSV is estimated to have vascular abnormalities on digital subtraction angiography in 69.3–97.8% (8, 9) of cases. This contributes to diagnostic delay.

The sparing of larger vessels accounts for the absence of large vessel stroke or haemorrhage. The presentation is rather characterised by subacute onset headache, motor and sensory deficits, seizures, and cognitive dysfunction. All existing cases report a seizure history, which relates to disease in small cortical or leptomeningeal vessels. In our series, one patient had onset of focal seizures 20 years prior that had been effectively treated since with lamotrigine. The relevance of this is uncertain; however, it could reflect a prodromal stage of disease as observed in Rasmussen’s encephalitis, another hemispheric inflammatory brain disease (10). Headache was common to all three of our patients and tended to recur with each relapse. Hemisensory and motor deficits with pyramidal weakness, and spasticity also occurred and demonstrated improvement with treatment.

Other than their lateralisation, UR-PCNSV lesions show a similar imaging pattern to typical PCNSV with irregular T2/FLAIR hyperintense lesions predominantly involving supratentorial white matter. Leptomeningeal and parenchymal enhancement is common and occurs in various patterns, including nodular, linear, and ring enhancement. In our series, some lesions were diffusion restricting. These sequences are inconsistently reported on in earlier cases, although Rezak et al. found no diffusion restriction in their seven patients, suggesting that microinfarction in UR-PCNSV is frequently below the resolution of diffusion-weighted imaging (1). Haemorrhage on SWI has been reported in up to 96.4% of PCNSV cases and is considered a supportive radiological finding (6). Susceptibility artefact was subtle in patients 2 and 3 and resolved on follow-up imaging. These patients underscore the risk of misdiagnosing PCNSV, as differentiating its lesions from CNS demyelination and neoplastic pathology can be challenging without biopsy. The presence of microhaemorrhage, even when minimal, should prompt further consideration of vasculitis.

Relapse activity is frequent in UR-PCNSV, and in this case series, monotherapy with an anti-CD20 monoclonal antibody was not adequate to maintain remission. All patients demonstrated recurrent or new lesion enhancement after steroid withdrawal. This is consistent with prior reports wherein prolonged prednisolone treatment at a daily dose of 20 mg or greater with additional immunosuppressive therapy was required to maintain remission (2). In addition to frequent relapses, patients demonstrated hemiatrophy within a year of diagnosis. This was reported in five earlier cases (1), none of whom received early high potency immunosuppression. Rasmussen’s encephalitis also causes progressive unilateral cerebral atrophy; however, onset is typically in children and histopathology is characterised by microglial nodules and neuronophagia (11). Gadolinium enhancing lesions and transmural vessel inflammation are not a feature of RE and differentiate the condition from UR-PCNSV (2). The high prevalence of cerebral atrophy early in UR-PCNSV is indicative of high inflammatory activity and reinforces the importance of prompt diagnosis and early high potency immunosuppression. A unilateral waxing and waning radiological course with incomplete resolution on therapy should raise suspicion of the diagnosis of UR-PCNSV on serial MRI.

A key take-away from this series is that patients did not achieve remission on anti-CD20 monotherapy, indicating that prolonged corticosteroids with additional immunosuppression are necessary. Rituximab has been proposed as an effective first-line therapy for PCNSV due to its success in retrospective cohort studies (12). Although the immunologic mechanisms underpinning PCNSV remain incompletely characterised, B cells have been implicated through their activation of complement, cytokine secretion, and antibody production (12). However, lymphocytic infiltrate in PCNSV is typically T-cell predominant (13), and b-cell depletion may be inadequate in patients with highly active diseases. It should be noted that the histopathology in our patients was impacted by earlier treatment with B-cell depleting therapy and no conclusions can be drawn from the absence of B cells in these specimens.

Several mechanisms underpinning the lateralising nature of UR-PCNSV have been proposed, although this remains poorly understood (2). It may be that local innate danger signals attract lymphocytes to specific vascular epitopes. Other immune mediated CNS diseases demonstrating lateralisation are Rasmussen’s encephalitis as well as unilateral cortical FLAMES (FLAIR-hyperintense Lesions in Anti-MOG-associated Encephalitis with Seizures) (11, 14). Hemispheric differences in gene expression have been found in the adult cortex (15), which is thought to arise from epigenetic regulation and could lead to differences in immune cell function and antigenic targets. Further work is needed to better elucidate this.

## Conclusion

UR-PCNSV is a rare subtype of PCNSV characterised by a younger age of onset, small-medium vessel involvement, high relapse frequency, and early risk of cerebral hemiatrophy. Headache, seizure, cognitive impairment, and hemi-sensorimotor deficits are common presenting features. Diagnostic delay is common, and a unilateral waxing and waning radiological course with incomplete resolution on therapy should raise suspicion for UR-PCNSV. Our series highlights that anti-CD20 mAb monotherapy is frequently ineffective, indicating the need for prolonged corticosteroid and/or alternative immunosuppressive therapy.

## Data Availability

The original contributions presented in the study are included in the article/**Supplementary Material**. Further inquiries can be directed to the corresponding author.
