# Supplementary material for: Case Report: Unilateral relapsing primary central nervous system vasculitis—expanding the phenotype
Source: Front Immunol. 2025 Jun 2;16:1502022. doi: 10.3389/fimmu.2025.1502022 (PMC12171174; doi:10.3389/fimmu.2025.1502022)
Supplement: Supplementary file 1 [file Table1.docx]

| Investigation | Patient 1 | Patient 2 | Patient 3 | Reference range |
| --- | --- | --- | --- | --- |
| **Serum** |  |  |  |  |
| Haemoglobin | 127 | 139 | 132 | 119-160 g/L |
| White cell count | 7.8 | 11.2 | 9.7 | 4.0-11.0 x10^9^/L |
| Platelet count | 294 | 223 | 215 | 150-450 x10^9^/L |
| Urea, electrolytes & creatinine | Normal | Normal | Normal |  |
| Liver function tests | Normal | Normal | Normal |  |
| C reactive protein (CRP) | 0.9 | 0.3 | 1.2 | 0.0-5.0 mg/L |
| Erythrocyte sedimentation rate (ESR) | Not tested | Not tested | 16 | 0.0-15 mm/hr |
| Anti-nuclear antibody (ANA) | Not detected | Not detected | Not detected |  |
| 3Anti-neutrophil cytoplasmic antibodies (ANCA) | Not detected | Not detected | Not detected |  |
| Extractable nuclear antigen (ENA) | Negative | Negative | Negative |  |
| Double stranded DNA (dsDNA) | Negative | Negative | Negative |  |
| Anti-neuronal antibodies* | Negative | Negative | Negative |  |
| Myelin oligodendrocyte glycoprotein antibody (MOG) | Negative | Negative | Negative |  |
| Aquaporin 4 antibody (AQP40 | Negative | Negative | Negative |  |
| Thyroid antibodies | Negative | Negative | Negative |  |
| HIV, hepatits B & C screen | Negative | Negative | Negative |  |
| Syphilis serology | Negative | Negative | Negative |  |
| Serum protein electrophoresis / protein immunofixation electrophoresis | Normal | Normal | Normal |  |
| **CSF (additional investigations)** |  |  |  |  |
| Viral PCR multiplex | Negative | Negative | Negative |  |
| Cytology | Normal | Normal | Normal |  |
| Flow cytometry | Normal | Normal | Normal |  |

*Anti-neuronal antibodies tested by immunofluorescence are PCA1, PCA2, ANNA-1, ANNA-2, Ma 1, Ma 2, CV2, amphiphysin, Tr and Sox-1.
